# Biphenotypic Sinonasal Sarcoma with Orbital Invasion: A Literature Review and Modular System of Surgical Approaches

**DOI:** 10.3390/cancers16193316

**Published:** 2024-09-27

**Authors:** Sergio Corvino, Oreste de Divitiis, Adriana Iuliano, Federico Russo, Giuseppe Corazzelli, Dana Cohen, Rosa Maria Di Crescenzo, Carmela Palmiero, Giuseppe Pontillo, Stefania Staibano, Diego Strianese, Andrea Elefante, Giuseppe Mariniello

**Affiliations:** 1 Department of Neurosciences and Reproductive and Odontostomatological Sciences, Division of Neurosurgery, School of Medicine, University of Naples “Federico II”, 80131 Naples, Italy; sercorvino@gmail.com (S.C.); oreste.dedivitiis@unina.it (O.d.D.); dr.federicorussonch@gmail.com (F.R.); giucoraz@gmail.com (G.C.); carme.palmiero@gmail.com (C.P.); giumarin@unina.it (G.M.); 2 Division of Ophthalmology, School of Medicine, University of Naples “Federico II”, 80131 Naples, Italy; cohen.dana19@gmail.com (D.C.); diego.strianese@unina.it (D.S.); 3 Department of Advanced Biomedical Sciences, Division of Pathology, School of Medicine, University of Naples “Federico II”, 80131 Naples, Italy; rosamaria.dicrescenzo@unina.it (R.M.D.C.); stefania.staibano@unina.it (S.S.); 4 Division of Neuroradiology, School of Medicine, University of Naples “Federico II”, 80131 Naples, Italy; giuseppe.pontillo@unina.it (G.P.); aelefant@unina.it (A.E.)

**Keywords:** biphenotypic sarcoma, endoscopy, endoscopic endonasal approach, transorbital approach, paranasal sinus tumors

## Abstract

**Simple Summary:**

Numerous different pathologies can primarily or secondarily affect the orbit. Among them, although rare in terms of incidence, biphenotypic sinonasal sarcoma should be considered. It is a low-grade tumor of the sinonasal tract with a tendency to invade the adjacent anatomical structures, especially the orbit and anterior skull base, accounting for potentially severe morbidities. Well-defined guidelines of treatment and surveillance protocols are lacking. Therefore, we perform a systematic literature review by analyzing the demographic, clinical, radiological, and treatment features, separately report a personal illustrative case, and discuss the surgical strategies, with the aim of shedding more light on this apparently benign pathology.

**Abstract:**

**Background:** Biphenotypic sinonasal sarcoma is a rare low-grade tumor arising from the sinonasal tract, featuring locally aggressive biological behavior, with a tendency to invade the orbit and skull base. There are no defined guidelines of treatment; thus, the management varies among different institutions. The aim of the present study is to provide a modular system of surgical approaches according to the lesion pattern of growth from a literature review. **Materials and Methods:** A comprehensive and detailed literature review on the PubMed and Embase online electronic databases on biphenotypic sinonasal sarcoma with orbital invasion was conducted. A personal case exhibiting peculiar features was also added. Demographic (patient’s sex and age), clinical (presenting symptoms and time to treatment), neuroradiological (anatomical origin and pattern of growth), and treatment (type of treatment, surgical approach, extent of resection, peri- and postoperative complications, and adjuvant therapies) data, as well as clinical outcome, recurrence rates, and overall survival, were analyzed. **Results:** Thirty-one patients harboring biphenotypic sinonasal sarcoma with orbital invasion were identified. Tumors mainly affected female patients (66.7%) and a middle-aged population (median 55.2 years old). Simultaneous skull base involvement occurred in most cases (80.6%). Surgery was performed in all but one case (97%), as unique treatment (59%) or in association with radio—(23.5%) and/or chemotherapy (5.9%/2.9%), allowing for gross total tumor resection in most cases (66.7%). The endoscopic endonasal approach was the most adopted surgical corridor (51.7%). The local recurrence rate was 19.3%, and only two cases of tumor-related mortality occurred. **Conclusions:** Surgery is the only curative treatment, with the main goal to restore/improve/arrest progression of clinical manifestations. The endoscopic endonasal route represents the master approach for lesions confined to the midline. Microsurgical transcranial and endoscopic transorbital approaches have a complementary role for addressing the lesion’s component with large intracranial extension or affecting the paramedian aspect of the anterior cranial fossa and superior–lateral orbital compartment, respectively. The approach selection should be made case by case according to the tumor pattern of growth.

## 1. Introduction

The orbit is a natural skeletal cavity communicating with exocranial and intracranial spaces through superior and inferior orbital fissures and the optic canal [1], and which can be primarily and/or secondarily affected by several neoplastic, infectious–inflammatory, vascular, and traumatic diseases [2,3,4,5,6]. Among them, although very rare in terms of incidence (1–5% of head and neck malignancies [7]), biphenotypic sinonasal sarcoma (BSNS) deserves to be considered. Introduced in the WHO classification of head and neck tumors in 2017 [8], BSNS is a low-grade tumor of the sinonasal tract, most commonly arising from the ethmoid or frontal sinus or nasal cavity, with a tendency to invade the orbit and/or skull base, usually through the cribriform plate and lamina papyracea, respectively. This pattern of growth accounts for the main presenting symptoms and signs, i.e., nasal obstruction and facial pressure, followed by epistaxis and orbital impairment [9].

Because of the paucity of data on the management of this tumor due to its relatively recent histological and molecular characterization, well-defined guidelines of treatment as well as a surveillance protocol are lacking. However, due to its anatomical origin close to highly functional structures, like the orbital content and the brain, and its locally aggressive and destructing pattern of growth, BSNS may account for severe and potentially irreversible neurological–ophthalmological deficits. Therefore, prompt and adequate management is imperative.

In this setting, first we attempted to make a detailed and comprehensive literature review on BSNS with orbital invasion, also providing a personal case, by analyzing demographic, clinical, and radiological features, as well as treatment and outcome data, to better define the natural course of this rare disease. In addition, as a secondary endpoint, we discussed the state of the art and enriched the current relevant knowledge, providing a modular system of surgical approaches for supporting more accurate management of this pathology.

## 2. Methods

**Figure 1:**
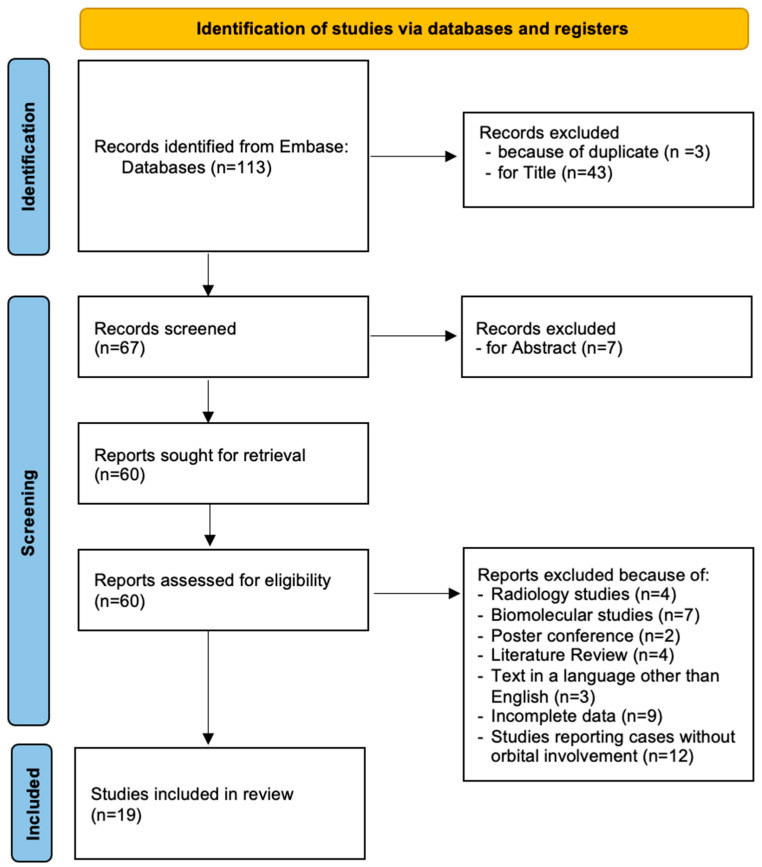
Flow chart showing the methods for the selection of the studies included in the review, following PRSIMA [10].

A Medline search from January 2012 to June 2024 in the Embase online electronic database was conducted in accordance with Preferred Reporting Items for Systematic Reviews and Meta-Analysis (PRISMA) guidelines [10], by using the following key sentences: “biphenotypic sinonasal sarcoma” OR “low-grade sinonasal sarcoma”, “biphenotypic sarcoma”, “frontal sinus”, “ethmoid sinus”, “maxillary sinus”, and “orbit”. They were combined as follows: (“biphenotypic sinonasal sarcoma” AND “orbit”), (“frontal sinus” AND “biphenotypic sarcoma”), (“ethmoid sinus” AND “biphenotypic sarcoma”), (“maxillary sinus” AND “biphenotypic sarcoma”), (“frontal sinus” AND “ethmoid sinus” AND “maxillary sinus” AND “biphenotypic sarcoma”), and (“frontal sinus” AND “ethmoid sinus” AND “orbit” AND “biphenotypic sarcoma”). After removal of duplicates, all abstracts were evaluated, and each article of interest was marked for further review. The full text of the marked studies was screened by two authors independently (S.C. and F.R.) and included in this systematic review following inclusion and exclusion criteria, as summarized in Figure 1.

The inclusion criteria encompassed surgical series, reviews, and case reports in the English language concerning BSNS with orbital involvement, with immunohistochemical diagnosis confirmed or not by molecular exams, and studies reporting relevant clinical and surgical data. Studies involving animals, duplicates, and studies about biphenotypic sarcomas of other localizations were excluded. Analyzed factors included the patient’s sex and age, presenting symptoms and signs, anatomical origin and pattern of growth, time to treatment, type of treatment, surgical approach, extent of resection, perioperative complications, recurrence, and overall survival.

### Statistical Analysis

Data were collected through an extensive examination of the described patients in the literature. Categorical and qualitative data were assessed through the Shapiro–Wilk normality test. The threshold for statistical significance was set at a *p*-value of 0.05. Data were aggregated in Microsoft Excel (version 14.2.5), and GraphPad software (version 10.2.2) was used to perform the analysis.

## 3. Results

### 3.1. Clinical Case

**Figure 2:**
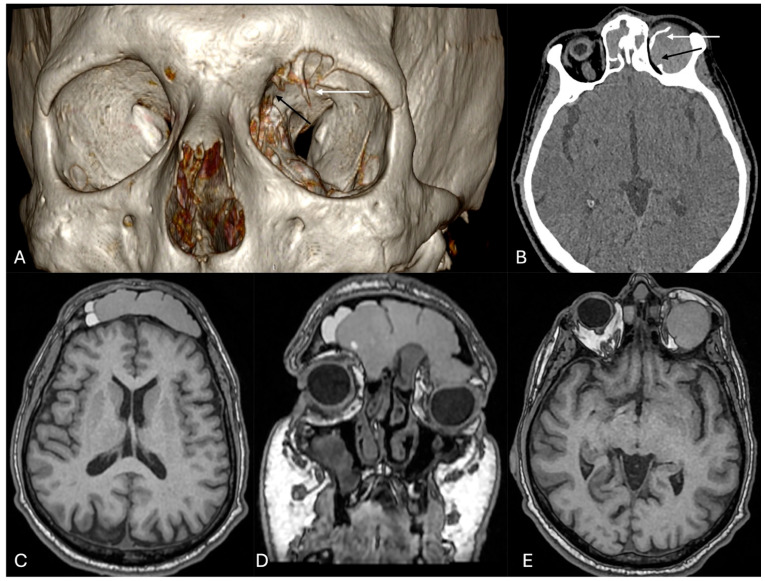
Preoperative diagnostic images. (**A**,**B**) Head CT scan: (**A**) 3D reconstruction and (**B**) axial sequence: a bony fragment protruding into the left orbital cavity (white arrows) and the bony erosion of the roof (black arrows) are evident; (**C**–**E**) contrast-enhanced brain MRI: axial (**C**), coronal (**D**), and axial (**E**) sequences: inhomogeneous contrast-enhanced lesion arising from the ethmoid sinus, occupying and occluding the frontal sinus (**C**,**D**), with extension into the left orbit (**E**).

A 46-year-old man complaining of a one-year history of visual acuity deficit and progressive proptosis in the left eye was observed. The neurological and ophthalmological assessment revealed, in the left eye, proptosis (grade I) with inferolateral displacement of the eyeball, associated to restriction in upward gaze. Head computed tomography (CT) showed a partially ossified lesion extending from the upper sinonasal tract until the frontal sinus and into the left orbit through its roof (Figure 2A,B). Head magnetic resonance imaging (MRI) showed an inhomogeneous contrast-enhanced lesion arising from the ethmoid sinus, occupying and occluding the frontal sinus, with extension into the left orbit through the erosion of its roof, compressing the superior obliquus and superior rectus muscles (Figure 2C–E).

These findings oriented toward a diagnosis of frontal sinus–orbital mucocele secondary to frontal sinus osteoma.

Patient underwent a combined one-stage left microsurgical transcranial fronto-basal and endoscopic endonasal approach. After craniotomy and gentle upward retraction of the homolateral frontal lobe, a large mucocele arising from the frontal sinus and extending into the left orbit was exposed; its capsule was incised and removed after draining its content. The left orbit was inspected following the corridor created by the lesion through the roof. The frontal sinus was opened and the lesion inside was completely removed; sinus mucosa was curetting out to “cranialize” the sinus at the end of the procedure. At that point, the endoscopic endonasal approach was performed to remove a small component of the lesion involving the ethmoid sinus to ensure the patency of the airway and frontal sinus drainage. The postoperative course was uneventful. A postoperative CT scan confirmed the lesion removal and the decompression of the left eyeball with resolution of the proptosis. At day 5 following the operation, the patient was discharged.

**Figure 3:**
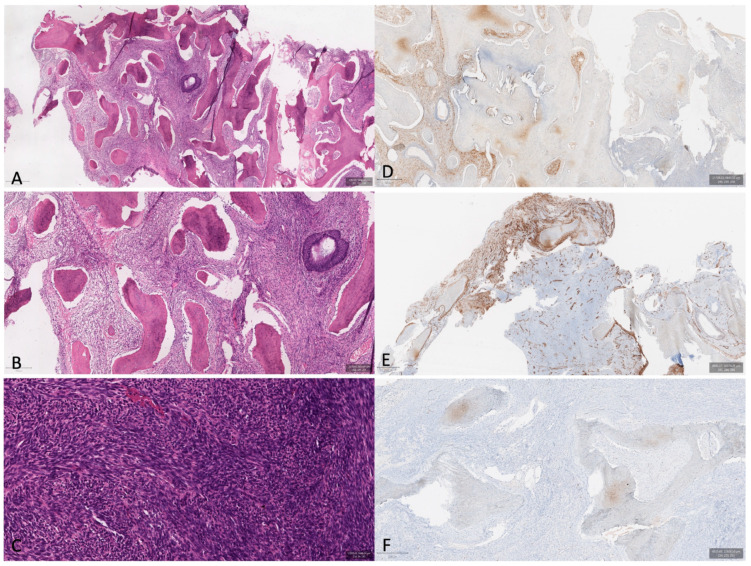
(**A**,**B**) Histological evaluation revealed an unencapsulated tumor, infiltrating bone tissue (H&E 10× and 20× respectively). (**C**) The tumor presented an infiltrative growth pattern and was composed of spindled cells forming medium-to-long fascicles, often with a herringbone pattern (H&E 20×). Immunohistochemical examination revealed focal positivity for both S100 (**D**) and actin (**E**). The Ki67 index was low, about 2% (**F**).

The histological and immunohistochemical studies documented high cellularity of spindle cells, featuring S-100 and smooth muscle actin (SMA) positivity, whereas SOX 10, CD34, and EMA were negative. The proliferation index assessed through Ki67-MIB1 was 2% (Figure 3).

The diagnosis was in favor of a sinonasal biphenotypic sarcoma complicated by frontal sinus mucocele and orbital invasion.

No adjuvant treatment was recommended and a clinical and neuroradiological follow-up at 6 months was suggested to the patient.

### 3.2. Literature Review

A detailed and comprehensive systematic literature review revealed 113 studies concerning biphenotypic sinonasal sarcoma. After removing duplicates and screening full texts of the marked studies included according to the inclusion criteria, 33 studies were identified [7,11,12,13,14,15,16,17,18,19,20,21,22,23,24,25,26,27,28,29,30,31,32,33,34,35,36,37,38,39,40,41]. After removing the reports including cases without orbital invasion, 19 studies were eligible for the review [7,16,18,19,21,22,23,24,25,26,29,30,31,32,35,36,37,38,39,40,41]. The entire sample included 31 patients.

All patients’ data are separately reported in Table 1 and Table 2 and summarized in Table 3 and Table 4.

### 3.3. Demographic, Clinical, and Neuroradiological Data (Table 1 and Table 3)

Date on gender was reported in 30 out of the 31 cases (96.7%), and 20 females (66.7%) and 10 males (33.3%), with a median age of 55.2 years (range 22–84 years old), were identified. Presenting symptoms was reported in 67.7% of cases (n = 21/31), and they were mainly represented by nasal obstruction (n = 14/21, 66.6%), followed by ocular impairment (n = 11/21, 52.4%)—including diplopia, epiphora, and gaze restriction—facial discomfort (n = 6/21, 28.5%)—including facial pain and/or pressure—and epistaxis (n = 2/21, 9.5%).

The anatomical origin of the lesion and its pattern of growth, detected on head imaging, were reported in 100% of cases. The most affected site was the ethmoid sinus (n = 26/31, 83.9%), followed by the nasal cavity (n = 22/31, 71%), frontal sinus (n = 14/31, 45.1%), maxillary sinus (n = 11/31, 35.4%), and sphenoid sinus (n = 1/31, 3.2%). From the site of origin, the lesion extended to the skull base (mainly anterior cranial fossa) in 80.6% of cases (n = 25/31).

### 3.4. Treatment and Outcome Data

The time to treatment was reported in 10 of the 31 cases (32.3%) and was 24 ± 48.5 months (mean ± SD). The type of treatment administered was reported in all but one study, including two patients operated on more than once, for a total number of 34 treatments. Among them, surgery was performed in all but one case (n = 33/34, 97%), where only the association of radio- and chemotherapy was administered (2.9%). In detail, surgical procedure was adopted as unique treatment in 20 patients (59%), while it was followed by adjuvant radiotherapy in eight cases (23.5%), by chemotherapy in one (2.9%), by both radio- and chemotherapy in two (5.9%), and in the form of biopsy in the other two (5.9%).

The description of the type of surgical approach selected was reported in 29 out of the 33 procedures (87.9%). The most adopted surgical corridor was the endoscopic endonasal route (n = 15/29, 51.7%), followed by the isolated microsurgical transcranial approach (n = 7/29, 24.1%) and the combined microsurgical transcranial–endoscopic endonasal approach (n = 6/29, 20.7%). An isolated transorbital approach was reserved only in one case (3.4%).

The extent of tumor resection was reported in 30 out of the 33 procedures performed (91%). It was the gross total (GTR) in 20 (66.7%) and the subtotal (STR) in the remaining 10 (33.3%).

In only twelve cases (36.4%) was the date concerning perioperative complications reported: they occurred in two patients (16.6%) and consisted of transient CSF leak (one) and infection of the pericranial flap and pneumocephalus (one).

Data on the recurrence rate were reported in all cases of the overall sample (n = 31/31, 100%). Among them, local recurrence was observed in six cases (19.3%). Finally, the follow-up was 50.48 ± 58.71 months (mean ± SD).

The status of 28 out of the 31 patients (90.3%) of the overall series has been reported at the last follow-up: twenty-five (89.3%) were alive and three had died. Two patients died due to tumor progression and one due to other causes.

## 4. Discussion

Due to the anatomical origin at the adjacent sinonasal tract, especially in the ethmoid sinus (83.9%), followed by the nasal cavity (71%), and due to the locally aggressive and destructing pattern of growth of the tumor, the orbital invasion as well as the skull base invasion seem to be part of the natural history of disease. Thirty-one cases of orbital involvement by BSNS have been identified in the present literature review, to which one case from our personal surgical series should be added, mainly occurring through invasion of the lamina papyracea and less frequently through the orbital roof. Simultaneous skull base involvement occurred in 80.6% of cases, mainly due to invasion of the cribriform plate. In this scenario, our personal case exhibited a distinctive feature, showed by only one other case reported in the literature review [24], i.e., the orbital invasion was not constituted by a tumoral component but a tumor-induced mucocele caused by obstruction of the frontal sinus ostium by the tumor.

Because of the proximity of the lesion to highly functional neurovascular structures, like the orbital content and the brain, and due to its locally aggressive biological behavior, which potentially account for irreversible neuro-ophthalmological deficits, a prompt and proper diagnosis as well as adequate treatment are imperative.

In this setting, surgery represents the mainstay of management, as it is performed in all but one case (97%), as unique treatment or in association with radio and/or chemotherapy, in the form of isolated or combined approaches, allowing gross total tumor resection to be achieved in most cases (66.7%), with a low rate of perioperative complications (16.6%). Several factors should be considered during the decision-making process of the treatment strategy, both related to the patient—including age, comorbidities, clinical symptoms and signs, quality of life, and life expectancy—and to the pathology—size, location, relationship with adjacent neurovascular structures, pattern of growth, and radiologic features.

### 4.1. Treatment Strategies

Due to the rarity of the pathology and the lack of well-defined guidelines of treatment, the prognostic role of the surgery and the extent of resection, as well as of the adjuvant treatments and re-surgery, are unknown [9]. Thus, some dutiful considerations spontaneously arise: To treat or not to treat asymptomatic patients? What should be the aim of treatment? When is the best time to perform surgery? When should adjuvant treatments be recommended?

The treatment could be based on the paradigm of a “symptom-oriented surgery” to avoid unnecessary surgical overtreatment; on the other hand, for lesions incidentally detected in asymptomatic patients, the treatment could be based on the paradigm of a “preventive surgery of associated complications”, or on a conservative “wait and see” strategy until symptoms occur. The management should be tailored to the patient, case by case.

Considering the biological behavior of the disease, characterized by its locally aggressive and destructive nature, with an invasive but not infiltrating pattern of growth—thus with a clear cleavage plane from the close anatomical structures—as well as tendency to locally recur (19.3%) and satisfactory rate of gross total resection (66.3%) associated with a low rate of perioperative complications, maximal safe tumor resection should be attempted, especially in young or middle-aged patients, with a long expectancy of life and good performance status. Conversely, in elderly patients, unnecessary overtreatment should be avoided and surgery should prioritize the other primary goals of treatment, which first include (1) ensuring the patency of the upper respiratory tract and drainage of the involved paranasal sinuses, then (2) resolution of the mass effect on the adjacent neurovascular structures, including orbital content, so as to arrest and/or prevent a further worsening of ophthalmological disturbances and aesthetic disfigurement, and finally (3) preventing related intracranial complications, like mucocele, CSF leak, pneumocephalus, meningitis, seizure, brain abscess, and subdural empyema. For these purposes, surgery represents the first line of treatment, and several approaches can be considered, including purely endoscopic or open microsurgical, endoscopic-assisted, or combined endoscopic–open microsurgical approaches, as single or multiportal corridors, in single or multiple stages, each with related pros and cons, based on the target area, the goal of surgery, pathology, and patient features.

### 4.2. Surgical Nuances

Extended endoscopic endonasal approaches (EEEAs) play a leading role for midline skull base pathologies spreading in the sagittal plane from the crista galli to the odontoid [42,43]. Due to the origin of the BSNS from the ventral midline anterior skull base and/or sinonasal tract—upper nasal cavity/ethmoid sinus/frontal sinus—this surgical route represents the first option for these lesions, providing a direct and short corridor to the target through a natural cavity such as the nose, by using a favorable angle of attack, with low morbidity, short hospital stay, avoiding scars, craniotomy, and manipulation of nervous structures. The most common intra-perioperative complication is the CSF leak, whose incidence is progressively decreased over the years along with the improvement of the skull base reconstruction techniques [44]. The main limit of EEEAs is represented by the lateral extension of the lesion in the coronal plane [45].

For lesions with prevalent involvement of the frontal sinus, like in our case, the standard techniques include Draf, nasofrontal, and Eloy approaches [46,47]. Nevertheless, the access to the lateral end of the frontal sinus still represents a challenge for EEEA, especially in cases with unfavorable or distorted anatomy, even with the DRAF III drill out approach [48]. Firstly, the lateral limit of endoscopic endonasal approaches was identified in a virtual sagittal plane passing through the lamina papyracea [49], subsequently in a plane 2 cm lateral to it, or in the mid-orbit meridian during trans-cribriform approach [45]; other authors [50] proposed the ratio of lateral tumor extension to intraorbital distance as the lateral limit. In this setting, in recent years, thanks to the continuous refinements of endoscopic instruments and techniques, including the orbital transposition [51], the limits of the frontal sinus surgery via endoscopic endonasal approach have been progressively expanded to different kinds of lesions localized more laterally in the frontal sinus [51,52,53]. However, the size and pneumatization of the frontal sinus vary among people, as well as the pattern of growth and consistency of the lesions, and to improve the extent of resection and the safety of the surgical procedure, it is important to expose the site of attachment as well as all the boundaries of the lesion.

In this scenario, the endoscopic transorbital approach (TOA) allows the limit of far lateral extension of the EEEA to be overcome, not only into the frontal sinus, but also into the superior and lateral compartments of the orbit and paramedian anterior skull base, providing a complementary surgical route. Since the introduction of the transorbital neuro-endoscopy surgery (TONES) concept by Moe et al. [54] in 2010, the indications of the endoscopic transorbital surgery for neurosurgical pathologies affecting the paramedian regions of anterior and middle skull base are rapidly expanding, thanks to the peculiar advantages of this approach [54,55,56,57,58,59,60,61,62,63,64,65]. Particularly, the endoscopic superior-eyelid transorbital approach (SETOA) adopts a coplanar pathway to the frontal sinus and anterior cranial fossa, avoiding the necessity of angled endoscopic instruments, and providing an optimal trajectory to the target not otherwise accessible via the transnasal corridor [66,67]. This route allows the management of the far lateral extension of the lesion from the midline, beyond the mid-orbit meridian, and affecting the most lateral part of the frontal sinus, orbit, and anterior skull base in a minimally invasive fashion, following the pathological corridor provided by the lesion or through the drilling of the orbital roof. The main postoperative complication is represented by the enophthalmos, whose incidence can be reduced with a proper orbital reconstruction [57,63]. The combined endoscopic endonasal and transorbital surgical strategy for frontal sinus lesions is largely demonstrated as safe and effective [67,68,69].

Finally, concerning the open microsurgical transcranial approach (TCA), the osteoplastic flap was the workhorse approach for pathologies limited to the frontal sinus before the popularization of the endoscopic techniques. Several transcranial approaches can be considered to address the component of BSNS invading the frontal sinus, anterior skull base, and the orbit, mono- or bilaterally, including bifrontal trans-basal, unilateral supraorbital, fronto-temporal craniotomies. These approaches can be combined to the endoscopic endonasal one to address the intracranial extension of the lesion, especially when bilateral involvement occurs [24,28,35,70].

Microsurgical transcranial, endoscopic endonasal, and transorbital approaches can be adopted in isolated or variously combined manner, according to the target and to the pathology and patient features. Therefore, during the preoperative planning, a careful and meticulous evaluation of the head CT scan to assess the boundaries of the frontal sinus is mandatory [71].

**Figure 4:**
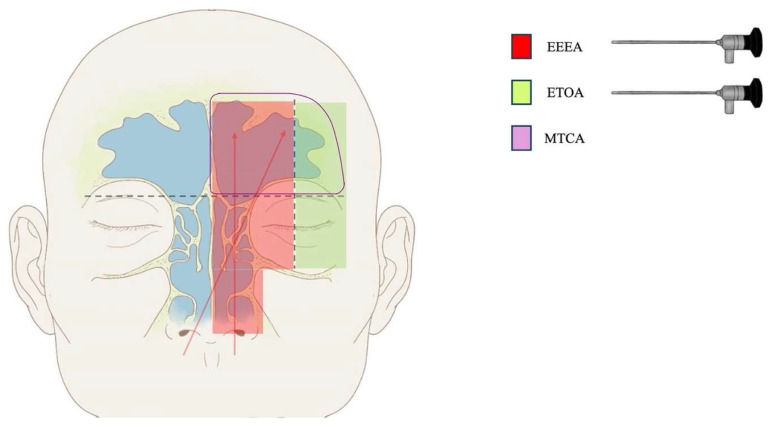
**Schematic diagram.** Blue perpendicular dotted lines define four quadrants: the superior–lateral and inferior–lateral (green areas), which can be accessed through ETOA (endoscopic transorbital approach), and superior–medial and inferior–medial (red areas), which can be accessed via EEEA (extended endoscopic endonasal approach). Finally, the transcranial approach is indicated for lesions involving the superior–medial and superior–lateral quadrants (purple line) bilaterally. Red arrows indicate the main far limits of the EEEA in approaching the frontal sinus.

Considering an imaginary vertical plane crossing the mid-orbit meridian and a perpendicular horizontal plane along the frontal sinus floor, the cribriform plate, and the orbital roof, we can ideally identify four compartments, which can be exposed and reached by tailored surgical approaches, defined according to the time (simultaneous or multistage) and the modality (isolated or variously combined) of performing as follows (Figure 4):

- Lesions affecting the superior–medial and inferior–medial areas, located medially to the mid-orbit meridian plane, and involving the upper nasal cavity, the ethmoid sinus, and the middle part of the frontal sinus can be accessed through an isolated EEEA (*single-port strategy*);
- The component of the lesion extending to the superior–lateral area, located laterally to the mid-orbit meridian and into the anterior cranial fossa, can be accessed through TCA or TOA (*two-port strategy: EEEA + TCA or EEEA + SETOA*);
- The component of the lesion extending to the inferior–lateral area, located laterally to the mid-orbit meridian and into the orbit, can be accessed through a TCA or TOA (*two-port strategy: EEEA + TCA or EEEA + SETOA*);
- The component of the lesion extending to the superior–lateral area bilaterally can be accessed through a bilateral TCA or TOA (*two-port strategy: EEEA + TCA, or three ports: EEEA + bilateral SETOA*).

Concerning our personal case, due to the origin of the lesion from the frontal and ethmoidal sinuses and the bilateral extension to the end of the lateral walls of the frontal sinus, we opted for a combined endoscopic endonasal and microsurgical transcranial approach, which allowed us to expose and attack the entire lesion.

The combination of these approaches provides a 360° exposure of the frontal sinus. A multiportal, combined, modular approach overcomes the limits of a single route in terms of exposure and working areas, providing multiple angles of attack from different perspectives and taking the advantage of the benefits offered by each single approach. Furthermore, the use of multiple “operative working angles” obviates the need for augmented “operative spaces”, avoiding the aggressive handling of the normal surrounding structures, extensive inner sinonasal disruption, minimizing the related comorbidities.

Obviously, along with the benefits, the risk of complications associated with each of approach is also increased. The selection of the surgical strategy should consider several aspects related to the patient, the pathology, and the goal of the treatment.

### 4.3. Limitation of the Study

The retrospective nature represents the first limit of the study. In addition, the small size of the sample of patients included and the heterogeneity of data represent other limitations of the study.

## 5. Conclusions

Biphenotypic sinonasal sarcoma exhibits peculiar biological and clinical features. To date, surgery represents the only curative treatment with the aim of restoring/improving/arresting the progression of symptoms and signs. In this setting, the endoscopic endonasal approach plays the leading role in addressing the main component of the tumor affecting the anterior midline structures, including the upper nasal cavity, ethmoid sinus, and median region of frontal sinus, with limited lateral extension. The tumoral component involving the far lateral wall of the frontal sinus, the upper and/or lateral compartments of the orbit, and in a small part the anterior skull base could be addressed via the endoscopic transorbital corridor, which provides an additional complementary port or through open microsurgical transcranial approach. If a large intracranial involvement occurs or the far lateral walls of the frontal sinus are bilaterally involved, a transcranial approach combined to the endoscopic endonasal route should be considered. The role of adjuvant therapies has yet to be determined.

## Figures and Tables

**Table 1:** Demographic, clinical, and radiological data of 31 cases of biphenotypic sinonasal sarcoma with orbital involvement.

	Authors/Year	Number of Cases	Sex, Mean Age (Years)	Presenting Symptoms	Anatomical Origin	Skull Base Involvement	Orbit Involvement
**1**	Cannon et al. [16] 2017	3	3 F (67.6 Years)	Diplopia, facial discomfort, nasal obstruction, facial pressure	3 FS-ES	3 YES	3 lamina papyracea
**2**	Hockstein et al. [41] 2018	1	F, 79	Asymptomatic	FS	YES	Roof
**3**	Koszewski et al. [40] 2018	1	M, 53	Unilateral nasal obstruction and epiphora	NC	YES (ACF)	Lamina papyracea
**4**	Chitguppi et al. [19] 2019	1	M, 53	n.a.	ES-NC	YES	YES
**5**	Alkhudher et al. [21] 2019	1	F, 35	Nasal obstruction, epistaxis	NC-MS-ES	None	Lamina papyracea
**6**	Miglani et al. [39] 2019	5	4 F, 1 M (56 years)	n.a.	5 NC-ES	5 YES (ACF)	5 lamina papyracea
**7**	Le Loarer et al. [22] 2019	4	3 F, 1 M (71 years)	n.a.	1 ES 1 ES-FS 1 NC-ES ES-FS	2 YES	4 YES
**8**	Kuhn et al. [38] 2019	1	n.a.	Worsening nasal obstruction, rhinorrhea, left orbital pain, proptosis, and blurry vision	NC-ES	YES (ACF)	Lamina papyracea
**9**	Okafor et al. [37] 2020	1	M, 54	Left-side nasal airway obstruction and anosmia	NC-MS-ES-FS	YES (ACF)	Lamina papyracea
**10**	Okuda et al. [36] 2020	1	F, 64	Nasal obstruction	NC-MS-ES pterygopalatine fossa	YES (MCF)	YES
**11**	Sethi et al. [23] 2021	2	2 F (56 years)	Nasal congestion and headaches	2 ES-MS-FS-NC	1 YES (ACF)	2 YES
**12**	Hanbazazh et al. [24] 2021	1	M, 50	Orbital pain and pressure, diplopia, blurred vision, lateral gaze restriction	ES	YES	Lamina papyracea
**13**	Bell et al. [25] 2022	1	M, 66	Swelling of left eyelid, vertical diplopia, and purulent nasal discharge	NC	YES (ACF)	YES
**14**	Hasnie et al. [26] 2022	1	F, 72	Nasal obstruction, episodic epistaxis and facial pressure/headaches, decreased sense of smell	MS-ES-Bilateral FS-NC	YES (ACF)	Lamina papyracea
**15**	Ingle et al. [29] 2023	1	F, 47	Swelling of the eyelid, proptosis	NC-FS-ES-MS	None	Lamina papyracea
**16**	Meyer et al. [30] 2023	1	M, 67	Nasal congestion and epiphora, right-side ocular proptosis	ES-MS-FS	None	YES
**17**	Kominsky et al. [31] 2023	2	2 M (65 years)	Bilateral nasal congestion and blurry vision	ES-NC-FS	2 YES	2 lamina papyracea
**18**	Bhele et al. [32] 2023	1	F, 22	Vision loss, headache, hyposmia, facial pressure	NC-ES-SS-MS	YES (ACF)	Lamina papyracea
**19**	Anastasiadou et al. [35] 2023	2	2 F (43 years)	Exophthalmos, headaches	NC-MS	1 YES	2 (1 floor, 1 lamina papyracea)

**M**: male; **F**: female; **n.a.**: not available; **ACF**: anterior cranial fossa; **ES**: ethmoid sinus; **FS**: frontal sinus; **SS**: sphenoid sinus; **MS**: maxillary sinus; **NC**: nasal cavity.

**Table 2:** Treatment and outcome data of 31 cases of biphenotypic sinonasal sarcoma with orbital involvement.

	Authors/Year	Number of Cases	Time to Treatment	Type of Treatment	Type of Surgical Approach	EOR	Peri- and Postoperative Complications	Recurrence	Status
**1**	Cannon et al. [16] 2017	3	n.a.	2 S 1 biopsy	1 EEA– 1 EEA + TCA 1 EEA Biopsy	2 GTR 1 STR	n.a.	1/3 (17 mo.)	(Mean 25 mo.) 3 alive
**2**	Hockstein et al. [41] 2018	1	12 mo.	S	EEA + TCA	GTR	n.a.	None	Alive
**3**	Koszewski et al. [40] 2018	1	4 mo.	S + Ad.RT	n.a.	STR	n.a.	None	Alive
**4**	Chitguppi et al. [19] 2019	1	n.a.	S + Ad-RT	TCA + ETOA	STR	n.a.	None	Alive
**5**	Alkhudher et al. [21] 2019	1	2 mo.	S	EEA	GTR	n.a.	None	Alive, 2 years
**6**	Miglani et al. [39] 2019	5	n.a.	4 S 1 S + Ad-RT	3 TCA 2 EEA	4 GTR 1 STR	n.a.	2/5 (mean 31.4 mo.)	(Mean 31.4 mo.) 5 alive
**7**	Le Loarer et al. [22] 2019	4	n.a.	1 CHT + RT 2 S 1 S + Ad.RT	n.a.	n.a.	n.a.	1/4 (after 91 mo.)	4 Alive (mean 176 mo.)
**8**	Kuhn et al. [38] 2019	1	n.a	S	TCA	GTR	None	n.a.	n.a.
**9**	Okafor et al. [37] 2020	1	5 mo.	2 S	2 EEA	1 STR 1 GTR	None	n.a.	n.a.
**10**	Okuda et al. [36] 2020	1	REC after 2 mo.	S + Ad.CHT	TCA	GTR	None	YES (after 2 mo.)	Dead after 8 mo. due to tumor progression
**11**	Sethi et al. [23] 2021	2	n.a.	1 S + Ad.RT 1 S	2 EEA	2 GTR	None	None	1/2 * alive (32 mo.)
**12**	Hanbazazh et al. [24] 2021	1	36 mo.	1 biopsy 1S 1S + Ad.RT	Biopsy EEA TOA TCA	3 STR	None	None	Alive
**13**	Bell et al. [25] 2022	1	REC after 15 years	1 S + Ad.RT	TCA	GTR	None	No further	Alive, 10 mo.
**14**	Hasnie et al. [26] 2022	1	24 mo.	S	EEA + TCA	GTR	Infection pericranial flap, pneumocephal	None	Death due to other causes
**15**	Ingle et al. [29] 2023	1	2 mo.	S	EEA + TCA	GTR	n.a.	None	Alive, 3 mo.
**16**	Meyer et al. [30] 2023	1	36 mo.	S + RT, CHT	EEA	STR	n.a.	Progression	Death after 15 mo. due to tumor progression
**17**	Kominsky et al. [31] 2023	2	3 weeks (1)	2 S	2 EEA	2 GTR	n.a.	None	2 alive (mean 13 mo.)
**18**	Bhele et al. [32] 2023	1	8 mo.	Neo-CHT, S, Ad-PB	TCA + EEA	STR	n.a.	None	Alive, 10 mo.
**19**	Anastasiadou et al. [35] 2023	2	n.a.	1S, 1S + Ad.RT	2 EEA	2 GTR	1 CSF leak	None	2 alive (mean 78 mo.)

* Available data; **mo.**: months; **n.a**.: not available; **GTR**: gross total resection; **STR**: subtotal resection; **S**: surgery; **RT**: radiotherapy; **CHT**: chemotherapy; **Ad**: adjuvant; **TCA**: transcranial approach; **EEA**: endoscopic endonasal approach.

**Table 3:** Summarized available demographic, clinical, and neuroradiological data of 31 cases of biphenotypic sinonasal sarcoma with orbital involvement.

Covariates	Overall Sample 31 (%)	Statistical Analysis (*p* Value)
**Demographic and clinical data**		
**Sex**	30/31 * (96.7%)	*p* = 0.66
- F	20/30 (66.7%)	
- M	10/30 (33.3%)	
**Age range** (Median)	22–84 years (55.2 years old)	S-W = 0.79; *p* = 0.04
**Main presenting symptoms**	21/31 * (67.7%)	*p* = 0.47
- Nasal obstruction	14/21 (66.6%)	
- Ocular impairment	11/21 (52.4%)	
- Facial pressure/pain/discomfort	6/21 (28.5%)	
- Epistaxis	2/21 (9.5%)	
**Radiological data**		
**Anatomical origin**	31/31 * (100%)	*p* = 0.23
- ES	26/31 (83.9%)	
- NC	22/31 (71%)	
- FS	14/31 (45.1%)	
- MS	11/31 (35.4%)	
- SS	1/31 (3.2%)	
**Skull base involvement**	31/31 * (100%)	*p* = 0.15
- Yes	25/31 (80.6%)	
- No	6/31 (19.4%)	

* Available data.

**Table 4:** Summarized available treatment and outcome data of 31 cases of biphenotypic sinonasal sarcoma with orbital involvement.

Covariates	Overall Sample 31 (%)	Statistical Analysis (*p* Value)
**Treatment data**		
**Time to treatment** (Mean ± SD)	10/31 * (32.3%) 24 ± 48.5 mo.	S-W = 0.52; *p* < 0.01
**Type of treatment**	34 *	*p* = 0.21
- S	20/34 (59%)	
- S + RT	8/34 (23.5%)	
- Biopsy alone	2/34 (5.9%)	
- S + CHT	1/34 (2.9%)	
- S + RT + CHT	2/34 (5.9%)	
- RT + CHT	1/34 (2.9%)	
**Type of surgical approach**	29/33 * (87.9%)	*p* = 0.13
- EEA	15/29 (51.7%)	
- TCA	7/29 (24.1%)	
- TOA	1/29 (3.4%)	
- Combined	6/29 (20.7%)	
**EOR**	30/33 * (91%)	*p* = 0.35
- GTR	20/30 (66.7%)	
- STR	10/30 (33.3%)	
**Peri- and postoperative complications**	12/33 * (36.4%)	*p* = 0.12
- Yes	2/12 (16.6%)	
- None	10/12 (83.4%)	
**Outcome**		
**Recurrence**	31/31 * (100%)	*p* = 0.6
- Yes	6/31(19.3%)	
- No	25/31 (80.7%)	
**Status**	28/31 * (90.3%)	*p* = 0.88
- Alive	25/28 (89.3%)	
- Dead	3/28 (10.7%)	
**Follow-up** (Mean ± SD)	50.48 ± 58.71	S-W = 0.66; *p* < 0.01

* Available data.

## Data Availability

Data of the current original research are available from the corresponding author on reasonable request.
